# Engineering Conductive Hydrogels with Tissue‐like Properties: A 3D Bioprinting and Enzymatic Polymerization Approach

**DOI:** 10.1002/smsc.202400290

**Published:** 2024-09-01

**Authors:** Changbai Li, Sajjad Naeimipour, Fatemeh Rasti Boroojeni, Tobias Abrahamsson, Xenofon Strakosas, Yangpeiqi Yi, Rebecka Rilemark, Caroline Lindholm, Venkata K. Perla, Chiara Musumeci, Yuyang Li, Hanne Biesmans, Marios Savvakis, Eva Olsson, Klas Tybrandt, Mary J. Donahue, Jennifer Y. Gerasimov, Robert Selegård, Magnus Berggren, Daniel Aili, Daniel T. Simon

**Affiliations:** ^1^ Laboratory of Organic Electronics Department of Science and Technology Linköping University 60174 Norrköping Sweden; ^2^ Laboratory of Molecular Materials Division of Biophysics and Bioengineering Department of Physics, Chemistry and Biology Linköping University 58183 Linköping Sweden; ^3^ Department of Physics Chalmers University of Technology 41296 Göteborg Sweden; ^4^ Bioelectronics Materials and Devices Lab Central European Institute of Technology Brno University of Technology Purkyňova 123 61200 Brno Czech Republic

**Keywords:** 3D printing, cell scaffold, conducting polymer, in vitro, polymerization

## Abstract

Hydrogels are promising materials for medical devices interfacing with neural tissues due to their similar mechanical properties. Traditional hydrogel‐based bio‐interfaces lack sufficient electrical conductivity, relying on low ionic conductivity, which limits signal transduction distance. Conducting polymer hydrogels offer enhanced ionic and electronic conductivities and biocompatibility but often face challenges in processability and require aggressive polymerization methods. Herein, we demonstrate in situ enzymatic polymerization of *π*‐conjugated monomers in a hyaluronan (HA)‐based hydrogel bioink to create cell‐compatible, electrically conductive hydrogel structures. These structures were fabricated using 3D bioprinting of HA‐based bioinks loaded with conjugated monomers, followed by enzymatic polymerization via horseradish peroxidase. This process increased the hydrogels’ stiffness from about 0.6 to 1.5 kPa and modified their electroactivity. The components and polymerization process were well‐tolerated by human primary dermal fibroblasts and PC12 cells. This work presents a novel method to fabricate cytocompatible and conductive hydrogels suitable for bioprinting. These hybrid materials combine tissue‐like mechanical properties with mixed ionic and electronic conductivity, providing new ways to use electricity to influence cell behavior in a native‐like microenvironment.

## Graphical abstract

This study introduces a novel method to enhance hydrogel conductivity and biocompatibility for biomedical applications. By using in situ enzymatic polymerization of *π*‐conjugated monomers within a hyaluronan‐based hydrogel bioink, followed by 3D bioprinting, the resulting hydrogels exhibit improved stiffness, electroactivity, and cytocompatibility. These conductive hydrogels provide a versatile platform for advanced 3D cell culture and neural engineering.

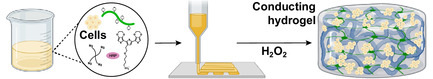

## 1 Introduction

Bioelectronic devices can provide powerful tools to efficiently communicate with electroactive neural cells and tissues, allowing us to better understand complex biological functions and treat patients suffering from neurological disorders.^[^ ^1^ ^]^ The cell or tissue interface of bioelectronic devices for neural applications can benefit from using synthetic hydrogels that match the mechanical and biochemical properties of tissue. Hydrogels mimicking the extracellular matrix are also widely used as cell‐supportive scaffolds in organ‐on‐chip devices,^[^ ^2^ ^]^ 3D cell culture,^[^ ^3^ ^]^ and as bioinks for 3D bioprinting.^[^ ^4^ ^]^ 3D bioprinting, which uses cell‐laden hydrogel‐based bioinks, enables the construction of more complex and functional tissue and disease models by arranging cells and materials with 3D spatial resolution, thus providing a paradigm shift in neural tissue engineering.^[^ ^4^, ^5^ ^]^

The incorporation of electroactive functionalities using conjugated polymer (CP)‐based materials in 3D bioprinted structures provides new means to create complex neural structures with the capability to stimulate or record neural activity.^[^ ^6^ ^]^ CPs such as polypyrrole, polyaniline, polythiophene, and poly(3,4‐ethylenedioxythiophene) (PEDOT) have recently gained considerable attention in the field of neural tissue engineering due to their ability to conduct charge through both electronic and ionic transport, bridging the signaling mismatch between electronics and biology.^[^ ^7^, ^8^, ^9^ ^]^ Additionally, the possibility to combine CPs with hydrogel networks makes them interesting for interfacing neural tissues with decreased mechanical mismatch and improved host responses. The tunable stiffness of cell‐interfacing materials is also of great importance for in vitro applications, such as 3D cell culture.^[^ ^10^ ^]^ The most widely used electroconductive hydrogels are based on PEDOT, resulting in conductive materials that are well‐tolerated by cells and tissues. While PEDOT is promising for in vitro studies of neuronal tissue, its use in tissue engineering is still limited by difficulties in material functionalization to improve the biological response, the absence of biodegradability, and poor reproducibility due to low solubility and processing challenges.^[^ ^11^, ^12^ ^]^

To address these issues, introducing water‐soluble *π*‐conjugated monomers that can be polymerized in situ can be considered as an attractive alternative to the more conventional approach of introducing pre‐polymerized CPs. After polymerization within the hydrogel, these materials typically exhibit electrical characteristics that are similar to those of other CPs but offer improved solubility and ease of processing while offering expanded possibilities for the introduction of diverse chemical functionalities^[^ ^13^, ^14^, ^15^ ^]^. Monomers are also better defined with respect to size and structure compared to CPs, which can improve the uniformity of their interactions with other materials as well as cells.^[^ ^16^, ^17^ ^]^ It has also been shown that short CPs (or oligomers) with less than 10 monomer units allow for material remodeling and degradation^[^ ^11^ ^]^ and can be eliminated by macrophages in vivo.^[^ ^18^ ^]^

**Figure 1:**
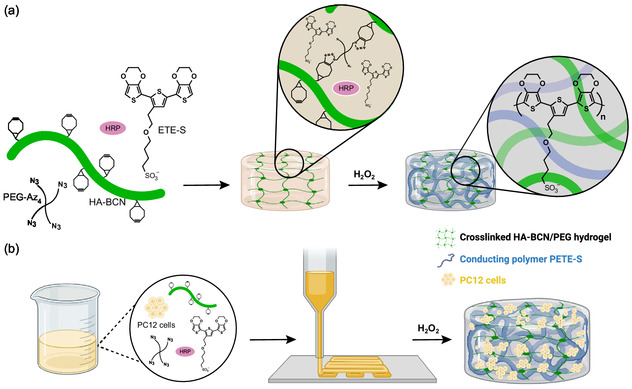
a) Schematic illustration of the formation of a conductive hyaluronan (HA)‐based hydrogel. HA‐BCN is cross‐linked using PEG‐Az_4_ in the presence of ETE‐S and HRP. The addition of H_2_O_2_ initiates the polymerization of PETE‐S and the formation of a conductive network within the hydrogel. b) Schematic illustration of bioprinting using HA hydrogel bioinks containing PC12 cells followed by in situ enzymatic polymerization of PETE‐S forming conductive pathways inside the HA‐BCN/PEG hydrogel. Illustration created with BioRender.com.

In this work, we demonstrate that it is possible to further functionalize hydrogel scaffolds by in situ enzymatic polymerization of the conjugated monomer sodium 4‐(2‐(2,5‐bis(2,3‐dihydrothieno[3,4‐b][1,4]dioxin‐5‐yl)thiophen‐3‐yl)ethoxy)butane‐1‐sulfonate (ETE‐S, **Figure** **1**a).^[^ ^19^ ^]^ The three‐ring ETE‐S “monomer” unit is comprised of a thiophene ring (modified with a 4‐ethoxy‐1‐butanesulfonic acid sodium salt side chain) with 3,4‐ethylenedioxythiophene (EDOT) moieties at 2,5‐position as two end groups (ETE = EDOT‐thiophene‐EDOT). The addition of the EDOT units provides the ETE‐S monomer with a lower oxidation potential for the conjugated system compared to single thiophene/EDOT monomers,^[^ ^20^, ^21^ ^]^ which allows gentler electrochemical polymerization, thus better interfacing with biological systems.^[^ ^19^ ^]^ This in situ enzymatic polymerization route to PETE‐S (or poly‐ETE‐S) does not require electrochemistry or strong redox chemistry and can even be modulated by endogenous metabolite concentrations, for example, in a physiological environment in the presence of PC12 cells, or in situ resulting formation of conductors within the structures of plant^[^ ^22^, ^23^, ^24^ ^]^ or animal tissues.^[^ ^25^ ^]^ Previous research has established that the integration of such CPs in soft materials can result in nanoscale topographies that facilitate intimate contact with cells.^[^ ^26^ ^]^

Here, we show a bioprintable, cytocompatible, and conductive hybrid hydrogel system fabricated by the integration of conjugated monomer‐based conductive nanoscale networks in hyaluronan (HA) hydrogel bioinks to mimic the seamless conductive network within native neural tissue (Figure 1b). HA is a biologically derived polysaccharide that is normally found in the extracellular matrix of vertebrates. HA can regulate cell proliferation, migration, and angiogenesis and is extensively used in biomedical applications, such as 3D cell culture,^[^ ^27^ ^]^ drug delivery,^[^ ^28^ ^]^ and regenerative medicine.^[^ ^29^ ^]^ We have previously developed modular hydrogel systems based on HA by conjugating bicyclo[6.1.0]non‐4‐yne (BCN) to the HA backbone (HA‐BCN) and cross‐linking with 4‐arm polyethylene glycol (PEG) with azides group conjugated at the terminal end of each arm (PEG‐Az_4_), resulting in HA‐BCN/PEG hydrogels using strain‐promoted azide‐alkyne cycloaddition (SPAAC) bioorthogonal click chemistry. Recently, HA‐BCN was used as a bioink for bioprinting of a functional 3D neural model comprising human long‐term neuroepithelial stem cells (lt‐NES) that could undergo spontaneous differentiation to neural fate.^[^ ^30^ ^]^ The further incorporation of PETE‐S in the HA‐BCN/PEG hydrogels via in situ enzymatic polymerization here results in the formation of a nanoscale conductive network. In addition to the tissue‐like mechanical properties and cytocompatibility of these hybrid materials, both electronic and ionic conductivity are displayed, facilitating the development of biocompatible neuro‐interfacing devices.

## 2 Results and Discussion

In this work, we have developed a printable, cytocompatible, and conductive modular hydrogel system based on in situ enzymatic polymerization of a conjugated monomer, sodium 4‐(2‐(2,5‐bis(2,3‐dihydrothieno[3,4‐b][1,4]dioxin‐5‐yl) thiophen‐3‐yl)ethoxy)butane‐1‐sulfonate (ETE‐S) in a bioorthogonally cross‐linked HA hydrogel. The HA hydrogel was obtained by first conjugating BCN to HA to obtain HA‐BCN. HA‐BCN was then cross‐linked using a 4‐arm PEG‐azide (PEG‐Az_4_) by a SPAAC reaction resulting in a HA‐BCN/PEG hybrid hydrogel as previously described.^[^ ^30^ ^]^ The SPAAC reaction between the BCN group on HA and the multi‐arm‐PEG‐azide occurs rapidly under physiological conditions without the needs of any additional compounds and is fully bioorthogonal allowing for efficient and tunable hydrogel formation.

To render conductive hydrogels, the ETE‐S monomers and horseradish peroxidase (HRP) enzyme were mixed with HA‐BCN prior to cross‐linking using PEG‐Az_4_. After cross‐linking of the hydrogels, the addition of H_2_O_2_ triggered HRP‐catalyzed polymerization of ETE‐S into PETE‐S in situ, seen as a drastic change in color from light brown to dark opaque over a period of 2 h (Figure S1b–d, Supporting Information). The polymerization mechanism of ETE‐S was investigated via ultraviolet‐visible (UV‐vis) absorption spectroscopy previously^[^ ^23^ ^]^ as well as in the solutions in this work and is in agreement with the change from light brown, corresponding to monomer peaks at 350 nm, to dark opaque, referring to the broad PETE‐S polymer band above 800 nm as shown in Figure S1a, Supporting Information. In the previous study, the involvement of H_2_O_2_ and peroxidase was investigated in the context of plant defense mechanisms suggesting they are the prime candidates for catalyzing the polymerization of ETE‐S without electrical input. The mechanism of ETE‐S polymerization was proposed following the classical scheme of the peroxidative cycle in plants starting from the HRP enzyme intermediate oxidized by H_2_O_2_, oxidizing one ETE‐S molecule forming one ETE‐S radical, followed by oxidative coupling between ETE‐S radicals to form larger ETE‐S oligomers. For our unpolymerized ETE‐S hydrogels, confocal fluorescence imaging showed that the ETE‐S monomers were distributed throughout the entire hydrogel matrix as shown by blue fluorescence in Figure S2, Supporting Information.

**Figure 2:**
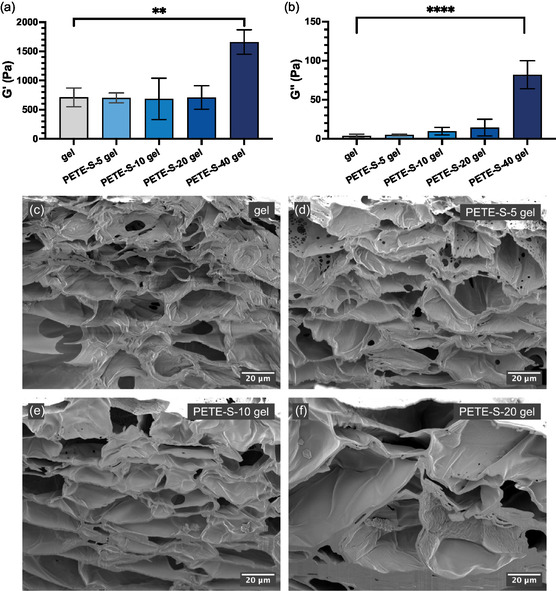
Material characterization of PETE‐S‐n gels. a) The storage modulus and b) loss modulus of gel samples (gel = HA‐BCN/PEG hydrogel) with varying amount PETE‐S (e.g., PETE‐S‐5 = PETE‐S from initial 5 mg ml^−1^ ETE‐S) after treatment in H_2_O_2_ for 2 h at room temperature. One‐way analysis of variance (ANOVA) with Dunnett's multiple comparisons test, ****P < 0.0001, **P = 0.0013, *n* = 3. SEM secondary electron images of cross‐sectioned freeze‐dried hydrogel samples showing the internal microstructure of c) clean gel (no PETE‐S), d) PETE‐S‐5 gel, e) PETE‐S‐10 gel, and f) PETE‐S‐20 gel. The images show the pore structure within the bulk materials. The sample surface is located at the top of the image for each sample.

This bioorthogonal hydrogel cross‐linking strategy is attractive for 3D neuronal cell culture because it is not detrimental to cell viability and can attain similar storage and loss modulus values to those of neural tissue (G′ ≈ 0.1–1 kPa).^[^ ^30^ ^]^ The presence of the ETE‐S prior to H_2_O_2_ treatment resulted in a brown hydrogel compared to the colorless control hydrogels (Figure S1b,c, Supporting Information). After the introduction of H_2_O_2_, the hydrogels immediately turned dark blue, indicating the formation of polymerized PETE‐S in the gel (Figure S1d, Supporting Information).^[^ ^25^ ^]^ The increasing concentration of PETE‐S increased both the stiffness of hydrogels and the loss modulus at 40 mg ml^−1^ (initial ETE‐S concentration), which shows the role of interpenetrated PETE‐S networks on the dissipation of energy applied to the gels (**Figure** **2**a,b). Note that in the figure and below, “gel” refers to the HA‐BCN/PEG hydrogel, and “PETE‐S‐*n*” refers to PETE‐S formed from an initial ETE‐S concentration of *n *mg ml^−1^. Thus “PETE‐S‐20 gel” refers to an HA‐BCN/PEG hydrogel functionalized by in situ polymerization from a 20 mg ml^−1^ ETE‐S solution, and “ETE‐S‐20” refers to the corresponding pre‐polymerized cocktail, that is, before addition of H_2_O_2_.

Scanning electron microscopy (SEM) secondary electron imaging of the hydrogels with and without PETE‐S revealed porous films (Figure 2c–f and Figure S3, Supporting Information). The pores are likely an effect of the lyophilization process but can also indirectly reveal differences in material properties. The structure can be described as pores separated by walls where the walls appear fibrous in morphology and not continuous. Pore sizes ranged from 1 to 100 μm with a trend of increasing pore size from 1 to 50 μm in the clean gel sample up to 20–100 μm in the PETE‐S‐20 gel. Pore walls in the hydrogels with PETE‐S were more continuous. As the monomer concentration increased from PETE‐S‐5 to PETE‐S‐20 (Figure S3b–d, Supporting Information), the fibrillar structure transformed into a more continuous morphology. As shown in Figure S3d, Supporting Information, the PETE‐S‐20 gel still showed small holes 0.5–5 μm in size in the pore walls. The number of these small holes decreased with increasing monomer concentration, suggesting that the interconnectivity and mass transport between pores also decreased.

The internal microstructure was revealed by cross sections prepared by focused ion beam (FIB) milling. SEM secondary electron imaging of these cross sections shows that the network of pores extends into the bulk structure (Figure 2c–f). The pore wall thickness increased when PETE‐S was added to the hydrogels, and the wall thickness increased with increasing concentration of the monomer. The pore wall thicknesses were 0.5–1.5 μm, 0.5–2.0 μm, 0.7–3.0 μm, and 1.0–10 μm for the clean gel, PETE‐S‐5 gel, PETE‐S‐10 gel, and PETE‐S‐20 gel, respectively. The distance from the surface also affected the microstructure, becoming more compact with increasing distance from the top surface since the wall thickness increased and the walls were more continuous.

It should be noted that the cross sections in Figure 2c–f show 2D slices of the 3D structures of the bulk material. The orientation of the pores and thereby the direction of the pore walls relative to the slicing direction may be different, resulting in a non‐orthogonal slicing of the pore walls. The 2D imaging provides a qualitative measurement of the difference in wall thickness between the samples with different monomer concentrations. A quantitative evaluation of the wall thicknesses requires 3D imaging. This would also reveal further information about the interconnectivity of the porous hydrogel network, as has previously been described.^[^ ^31^, ^32^, ^33^ ^]^

**Figure 3:**
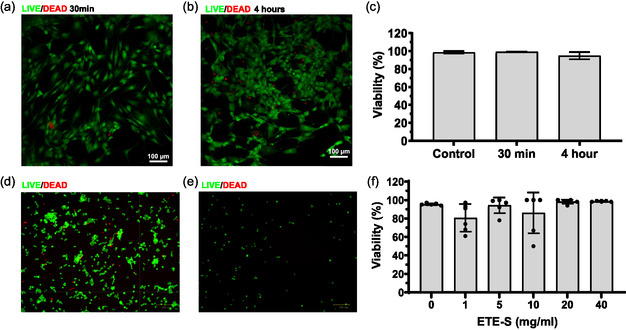
Microscopy images show live (green) and dead (red) fibroblast cells after exposure to 5 mg ml^−1^ ETE‐S after a) 30 min and b) 4 h. c) Cell viability reported as proportion of living cells at each time point and compared with control samples that were not exposed to ETE‐S. One‐way ANOVA with Dunnett's multiple comparisons test, not significant, *n* = 3 replicates for each group. d) PC12 cells incubated for 30 min with 0 mg ml^−1^ ETE‐S and e) 10 mg ml^−1^ ETE‐S. f) Cell viability reported for cells incubated for 30 min with 0, 1, 5, 10, 20, and 40 mg ml^−1^ of ETE‐S. Analysis by one‐way ANOVA with Dunnett's multiple comparisons test determined the variation of the means to be not significant, *n* = 6 replicates for each concentration.

To investigate the compatibility of ETE‐S for cell culture applications, we conducted an experiment in which human primary fibroblast cells and PC12 cells were cultured on tissue culture plates and incubated with ETE‐S. The results showed that fibroblasts maintained high viability after 30 min and 4 h of incubation with 5 mg ml^−1^ ETE‐S (**Figure** **3**a–c). Additionally, PC12 cells tolerated the presence of ETE‐S at concentrations of 0, 1, 5, 10, 20, or 40 mg ml^−1^ in cell culture medium for 30 min (Figure 3d–f). However, significant differences in total cell numbers were observed between control samples containing 0 mg ml^−1^ ETE‐S and treated samples with the varied concentrations of 1, 5, 10, 20, and 40 mg ml^−1^ ETE‐S (Figures 3d,e and S4, Supporting Information).

**Figure 4:**
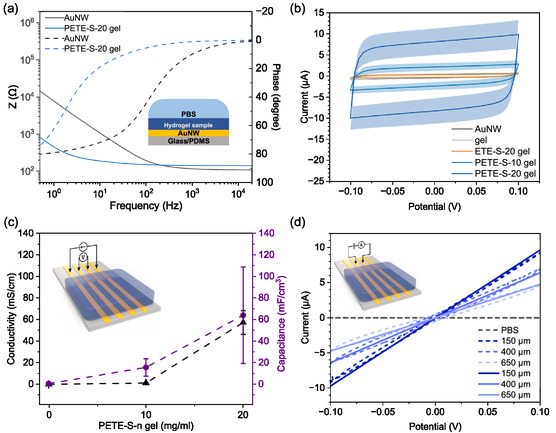
Stable and conductive PETE‐S gel formed after the addition of H_2_O_2_. a) EIS of AuNW (gray) and PETE‐S‐20 gel (blue), electrodes dimension *D* = 4 mm (*n* = 1 representative EIS measurement). b) Cyclic voltammograms of AuNW (black), gel (gray), ETE‐S‐20 gel (orange), PETE‐S‐10 gel (blue), and PETE‐S‐20 gel (dark blue) in PBS buffer solution (10 mM, pH 7.4) within the potential window –0.1 to 0.1 V at 50 mV s^−1^ (*n* = 3 individual measurements). c) Conductivities (left) of dry gel, dry PETE‐S‐10 gel, and dry PETE‐S‐20 gel and capacitances (right) of gel, PETE‐S‐10 gel, and PETE‐S‐20 gel as a function of initial ETE‐S monomer concentrations (*n* = 3 individual measurements). d) Current‐voltage measurements from PETE‐S‐20 gel on two AuNW electrodes with spacing distances of 150, 400 μm, or 650 μm in wet (dash line) and dry states (solid line). The application of a linear voltage sweep was from –0.1 to 0.1 V with a step of 10 mV (*n* = 1 representative current‐voltage measurement).

Enzyme‐assisted polymerization of ETE‐S forms mixed ionic‐electronic conductive hydrogels, as was demonstrated by the impedance change through the conductive hydrogel. **Figure** **4**a shows the electrochemical impedance spectra for a gold nanowire (AuNW) electrode and a AuNW electrode coated by a conductive PETE‐S‐20 gel with identical working area dimensions. At high frequencies, a flat impedance response is observed, which reflects the resistance of the electrolyte.^[^ ^34^ ^]^ The slight increase in PETE‐S‐20 impedance at high frequency is likely due to the increase in charge transfer resistance at the interfaces. As the frequencies decrease, the impedance increases due to capacitive behavior attributed to the ionic double layer at the interface. In the low frequency regime (≤100 Hz), as seen in the Bode plot in Figure 4a, we observed a striking drop of the impedance magnitude with a phase shift toward lower frequencies for PETE‐S‐20 gel compared to a pristine AuNW electrode (Figure 4a). By incorporating PETE‐S inside the hydrogel matrix, we were able to achieve decreased electrochemical impedance with the lowest impedance obtained for the PETE‐S‐20 gel. The decreased impedance can be attributed to the electrical connection of the thick PETE‐S gel film with the AuNW electrode. To better understand the charge capacity of the optimized conductive hydrogel, we compare the cyclic voltammograms of bare AuNW electrodes in a hydrogel containing no ETE derivatives to those of AuNW electrodes modified by PETE‐S‐20 gel, PETE‐S‐10 gel, and ETE‐S‐20 gel (Figure 4b). The capacitance currents of PETE‐S gels are much higher than those of the nonconductive gel modified AuNW electrodes and bare AuNW electrodes, which indicates that capacitive processes governed the charge interaction at the interface due to the larger electrically conductive surface area obtained for conductive gel modified AuNW electrodes. This observed phenomenon supported the higher volumetric capacitance of conductive hydrogels endowed by enzymatically polymerized PETE‐S, with the highest value of 63.89 mF cm^−3^ obtained for PETE‐S‐20 gel (Figure 4b).

Polymerization was monitored in situ as an increase in the current between two electrodes connected by the hydrogel (as in the inset in Figure 4d). The polymerization process is complete within 2 h of the addition of H_2_O_2_ (Figure S5a, Supporting Information), after which, film conductivity was measured in the dry state. The highest conductivity value of 57 mS cm^−1^ was obtained for PETE‐S‐20 gel (Figure 4c). The PETE‐S‐20 gel was further characterized using a two‐point configuration on an AuNW multi‐probe device. The application of a linear voltage sweep resulted in a linear current response consistent with Ohm's law, displaying a marked increase in current when compared to a control with only the electrolyte. The maximum observed current was 9.73 μA in the dry state and 9.31 μA in the wet state when –0.1 V was applied (Figure 4d).

We observed an enhancement in both conductivity and capacitance with increasing initial concentration of ETE‐S up to 20 mg ml^−1^. The conductivity assessments may not entirely capture the maximum capabilities of the conductive hydrogels, as their conductivity could potentially be enhanced through additional electrochemical doping. To investigate this further, we acquired the output and transfer characteristics of organic electrochemical transistors (OECTs) constructed with the conductive hydrogels as the active channel materials, as illustrated in Figure S5b, Supporting Information. From the transfer curve, it can be seen that the drain current (*I*_D_) increased by a factor of 4 as the gate voltage shifted toward negative values, indicating that the gels are not completely doped in their intrinsic state (Figure S5c,d, Supporting Information). The conductive PETE‐S‐20 gel‐based OECT exhibited a current of around –60 μA for a drain voltage (*V*_D_) of –0.6 V and a gate voltage (*V*_G_) of –0.4 V as well as a maximum transconductance of 0.13 mS.

**Figure 5:**
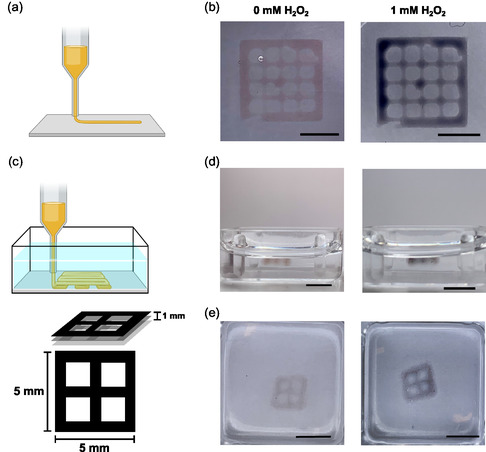
a) Schematic illustration of printing 2.5D structures. b) Printed 10 × 10 × 0.3 mm structure after 30 min incubation at room temperature in PBS buffer solution (10 mM, pH 7.4) and immediately after treatment with 1 mM H_2_O_2_. c) Schematic illustration of printing HA‐BCN/PEG/ETE‐S bioink inside gelatin support bath and the schematic presentation of 5 × 5 × 1 mm lattice. d) Side view of the printed structure after 30 min of gelation at room temperature and after removing the gelatin support bath, (left) before and (right) 30 min after adding 1 mM H_2_O_2_. e) Top view of the printed lattice in gelatin before and 30 min after adding 1 mM H_2_O_2_ to the PBS buffer. (Scale bars = 5 mm).

We proceeded to explore the possibilities of printing 3D hydrogel structures using a bioprinter and initiating in situ ETE‐S polymerization post‐printing. First, we printed a 2.5D (i.e., vertically thin) 10 × 10 × 0.3 mm lattice using a HA‐BCN bioink containing PEG‐Az_4_ and ETE‐S in air (**Figure** **5**a). The printed single‐layer structures were incubated at room temperature for 30 min. Post‐gelation, the samples were treated with H_2_O_2_, resulting in a visible color change to dark blue, indicating the formation of conductive PETE‐S. Samples treated with phosphate buffered saline (PBS) buffer instead of H_2_O_2_ solution did not change color (Figure 5b). In situ ETE‐S polymerization was further investigated by printing the ink into a gelatin support bath. The support bath enables the printing of soft bioinks and enhances the shape fidelity of deposited inks by acting as a temporary support during hydrogel cross‐linking (Figure 5c). Using the free‐formed reversible embedding of suspended hydrogels (FRESH) printing technique,^[^ ^35^ ^]^ it is possible to print complex geometries using HA‐BCN‐based bioinks.^[^ ^27^ ^]^ After 1 h incubation of the printed 5 × 5 × 1 mm lattice at room temperature, the gelatin support bath was melted by heating to 37 °C. The printed structures were then washed using warm PBS. The color of the printed structure changed as expected upon the addition of H_2_O_2_, again confirming the formation of the conductive PETE‐S (Figure 5d,e). These data demonstrate the printability of HA‐BCN/PEG/ETE‐S bioink and post‐print polymerization of ETE‐S.

**Figure 6:**
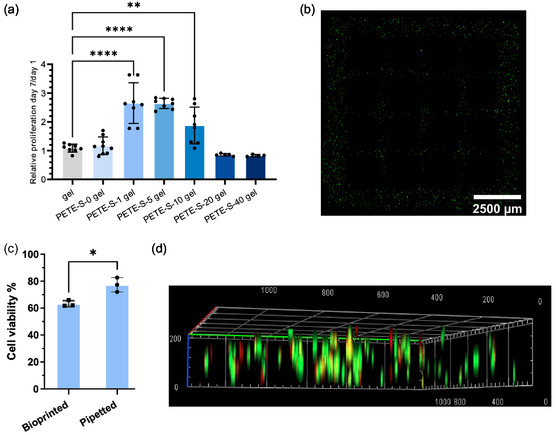
a) Relative cell proliferation ratios between day 7 and day 1 for PC12 cells (seeding density: 10^6^ cells ml^−1^) embedded inside control gel and PETE‐S‐0, −1, −5, −10, −20, and −40 gel, assessed using alamarBlue assay. Data are presented as means ± SD. One‐way ANOVA analysis and Dunnett's multiple comparisons test were used, **P = 0.0027, ****P < 0.0001 (*n* = 4–9 replicates for every group). b) Live/dead assay after 24 h incubation in 3D bioprinted structure using PETE‐S‐1 gel bioink, 10 × 10 × 0.3 mm lattice structure with 20% infill density, same pattern from Figure 5b; note the 4 × 4 grid of dark voids with no cells. c) PC12 cell viability encapsulated inside PETE‐S‐1 gel 24 h after bioprinting and pipetting, analyzed using live/dead assay (unpaired t‐test, *P = 0.0137, *n* = 3 replicates for each group). d) Confocal microscopy image of 3D distribution of live (green) and dead (red) cells in bioprinted PETE‐S‐1 gel after 24 h (unit: μm).

To investigate the cytocompatibility of the hydrogels and the conductive component PETE‐S, we encapsulated and cultured PC12 cells for a period of 7 days. PC12 cells were first mixed with HA‐BCN hydrogel precursor solution containing equal amounts of ETE‐S monomer and HRP. Cross‐linking was started by the addition of PEG‐Az_4_, followed by post‐polymerization of ETE‐S initiated by H_2_O_2_, all in the presence of the embedded PC12 cells. Control gels without cells were also compared to gels without cells incorporating 20 mg ml^−1^ ETE‐S monomers (ETE‐S‐20 gel), showing that the alamarBlue assay signals were not influenced by the ETE‐S monomers themselves (Figure S6a, Supporting Information). Additionally, control gels containing cells were compared to PETE‐S‐0 gels (i.e., same protocol for other PETE‐S‐n gels including H_2_O_2_ posttreatment, but 0 mg ml^−1^ ETE‐S) to exclude the effect of H_2_O_2_ on cell proliferation following a 7‐day culture period (Figure S6a, Supporting Information). After 7 days of cell culture, the cell‐laden hydrogel solutions were first collected and evaluated using the alamarBlue proliferation assay, confirming the absence of contaminants that could skew the results (Figure S6b, Supporting Information). PC12 cells showed significantly improved cell proliferation for PETE‐S‐1 gel (i.e., HA‐BCN/PEG/PETE‐S hydrogel with initial ETE‐S concentration 1 mg ml^−1^), PETE‐S‐5 gel, and PETE‐S‐10 gel on day 7 compared to day 1, evaluated by alamarBlue assay compared to control gels (**Figure** **6**a). The low proliferation observed in normal gel may be due to the preference of PC12 cells to proliferate in suspension, as there are no adhesion motifs for PC12 cells to attach to, which is a prerequisite for their proliferation in normal gel. Incorporating an intermediate concentration of ETE may help create larger pores in the internal structure and/or enhance the interaction between conducting polymers and PC12 cells, thereby facilitating proliferation. The PETE‐S‐20 gel and PETE‐S‐40 gel showed a less favorability for cell proliferation, which could be explained by the increasingly compact structure of the hydrogel as the monomer concentration increases, as was observed in Figure 2d–f, and by the drastic increase in stiffness (Figure 2a) and the toxicity of long monomer exposure time before consumed for polymerization. The compact structure may hinder nutrients from reaching the cells inside the hydrogel matrix.

To assess the possibility of printing cell‐containing conductive hydrogel as a bioink, hydrogel lattice structures (10 × 10 ×0.3 mm) were fabricated using a CELLINK BIO X bioprinter. Bioinks containing HA‐BCN, PEG‐Az_4_, 1 mg ml^−1^ ETE‐S, 1 mg ml^−1^ HRP, and PC12 cells were prepared in a cartridge and extruded by applying 20 kPa pressure through a 25 G needle on a 24‐well plate. After printing, H_2_O_2_ prepared in RPMI 1640 medium was added to the self‐standing and cross‐linked hydrogel lattice structure after a 30‐min gelation window by storing it in a sealed and moisture‐rich environment. We compared the cell viabilities (live/dead assay) at 24 h both for bioprinted and pipetted samples, that is, two deposition methods for the same bioink. PC12 cells embedded in bioprinted structures showed reduced cell viability of 63% compared to 77% for pipetted samples (Figure 6c). This suggests PC12 cells were exposed to substantial shear forces, which could be detrimental to viability, regardless of conductive component formation via H_2_O_2_ addition. To address this issue, alternative printing strategies, such as the FRESH technique or using nozzles with larger diameters, can be potential solutions. Meanwhile, by encapsulating the cells inside a conductive hydrogel bioink, they were provided a more uniform spatial distribution in a larger volume compared to the drop‐cast cell‐laden conductive hydrogel prepared by pipetting. The interconnected structures were first designed by computational modeling and then fabricated by bioprinter to ensure that each segment is proximal to nutrient sources, thus overcoming the limitations of nutrient and oxygen transport compared to bulky samples prepared using pipetting (Figure 6b,d).

## 3 Conclusions

In this work, we have developed a robust strategy to electrically functionalize click‐chemistry‐cross‐linked HA‐BCN/PEG hydrogels using enzymatically driven polymerization of the conducting polymer PETE‐S. We successfully fabricated electroactive scaffolds of complex architectures combining the advantageous physical properties of HA‐BCN/PEG hydrogels and the electronic functionality of the conducting polymer PETE‐S. The sulfonate group of the hydrophilic conjugated monomer ETE‐S facilitated a more homogeneous incorporation of conductive polymer networks within the hydrophilic HA‐BCN/PEG hydrogels, and the distributed PETE‐S affected both the electrical and mechanical characteristics of the resulting conductive hydrogels. Our modular approach allows the addition of conductive properties after the initial scaffold fabrication, enabling visual monitoring of cell behavior in the transparent scaffold's biochemical environment before introducing electrical and ionic conductivity. This allows precise control over the timing of introducing conductivity with minimal interference with cellular processes. Notably, the polymerization of ETE‐S into PETE‐S employs a mild enzymatic polymerization, which does not require electrochemistry or strong redox chemistry and can be regulated in situ by adjusting metabolite concentrations in the physiological environment of PC12 cells. This strategy opens new avenues for developing biocompatible and conductive hydrogel scaffolds that can be controlled dynamically, for example, by using endogenous signals to trigger polymerization. Significantly, we demonstrated the suitability of these conductive hydrogel scaffolds used for 3D cell culture promoting PC12 cell proliferation in a 7‐day culture period without external electrical stimulation. Additionally, the conductive HA‐BCN/PEG/PETE‐S hydrogel scaffolds fabricated using 3D bioprinting offer a powerful tool to develop more physiologically relevant models in vitro. 3D cell‐laden scaffolds fabricated by bioprinting overcome the limitations of nutrient and oxygen transport within pipetted samples, although we observed decreased cell viability attributed to substantial shear forces during bioprinting. Our results demonstrate that HA‐BCN/PEG/PETE‐S conductive hydrogels could be considered as a promising material for designing advanced biomimetic 3D cell culture platforms for neural engineering applications. Future efforts will aim to gain a deeper understanding of how these conductive hydrogels can be leveraged within the field of regenerative tissue engineering, particularly in 3D cell culture applications involving electrical recording or external electrical stimulation.

## 4 Experimental Section

4.1

4.1.1

##### Materials

Hyaluronic acid (100–150 kDa) was purchased from Lifecore Biomedical (Minnesota, USA). RPMI 1640 media with L‐glutamine, supplemented with 10% fetal bovine serum (FBS) and 5% heat‐inactivated horse serum (HS), alamarBlue Cell Viability Reagent (cat. no. DAL1025), as well as the LIVE/DEAD viability kit (cat. no. L3224), were obtained from Fisher Scientific. PEG‐Az_4_ (10 kDa) was purchased from Creative PEGworks, USA. Dialysis membrane (MWCO 12‐14 kDa) was acquired from Spectra/Pro RC, Spectrum Laboratories Inc. DMEM F12 media were purchased from VWR and supplemented with 10% FBS (Sigma‐Aldrich). H_2_O_2_, HRP (type I), PBS, N‐(3‐dimethylaminopropyl)‐N′‐ethylcarbodiimide (EDC), 1‐hydroxybenzotriazole hydrate, N‐[(1 R,8S,9s)‐Bicyclo[6.1.0]non‐4‐yn‐9‐ylmethyloxycarbonyl]‐1,8‐diamino‐3,6‐dioxaoctane (BCN‐NH_2_), poly‐L‐lysine (PLL) solution (P8920), and 2‐(N‐morpholino)ethanesulfonic acid (MES) (100 mM, pH 7) were purchased from Sigma‐Aldrich.

##### Material Synthesis: ETE‐S

A thorough description of ETE‐S synthesis has been previously reported.^[^ ^14^ ^]^

##### Material Synthesis: HA‐BCN

HA‐BCN was synthesized as described previously.^[^ ^30^ ^]^ Briefly, HA was dissolved in MES (100 mM, pH 7), and BCN‐NH_2_ was dissolved in 5:1 v v^−1^ acetonitrile: water solution. After the addition of EDC and 1‐hydroxybenzotriazole hydrate to the BCN‐NH_2_ mixture, it was added to the HA solution and allowed to react for 24 h at room temperature. The product was dialyzed (MWCO 12–14 kDa, Spectra/Pro RC, Spectrum Laboratories Inc.) against acetonitrile: water (1:10 v v^−1^) for 24 h followed by dialysis against MilliQ water for 3 days. The dialyzed sample was then lyophilized to a dry powder. The derivatization degree was estimated at 19% based on H^1^NMR.

##### Gold Nanowire Electrode Fabrication

AuNWs were synthesized by adopting a previously reported method.^[^ ^36^ ^]^ AuNW dispersion was vacuum filtered through a patterned polyvinylidene difluoride (PVDF) membrane to form a uniform and dense AuNW layer of 1 mg cm^−2^ areal coverage. Polydimethylsiloxane (PDMS, Dow Corning Sylgard 184) was spin‐coated (1000 rpm spin speed, 60 s) onto a glass wafer and semi‐cured (70 °C, 10 min). The dried PVDF membrane containing the AuNWs was placed on the semi‐cured PDMS under pressure (at 70 °C for 10 min), and the PVDF membrane was peeled off after being soaked in distilled water, followed by baking at 70 °C for 3 h for curing of the PDMS. A MetaQuip laser ablation system with a 355 nm pulsed UV laser was used to engraving the AuNWs to attain the designed electrode dimensions. The PDMS encapsulation layers were spin‐coated, and 4 mm opening circles were masked off to define the working area of AuNW electrodes for contacting hydrogel samples for electrical measurements.

##### Enzymatic Polymerization of ETE‐S in Solution: UV‐Vis Absorption Spectroscopy

Stock solutions of H_2_O_2_, ETE‐S, and HRP in PBS buffer (10 mM, pH 7.4) were freshly prepared before UV‐vis spectroscopy measurements. Absorption spectra were measured in a microplate reader (BioTek, Synergy H1). The final concentrations were 0.1 mg ml^−1^ (176 μM) for ETE‐S and 0.1 mg ml^−1^ for HRP (≥50 U mg^−1^). The amount of H_2_O_2_ corresponding to 1.5 equivalent versus ETE‐S was used to initiate the enzymatic polymerization. The spectra were recorded 2 h after mixing with a 1‐min premixing step.

##### Formation of Conductive Hydrogels

A solution of HA‐BCN (20 mg ml^−1^) was prepared in PBS (10 mM, pH 7.4). Nonconductive hydrogels were prepared by mixing HA‐BCN with an aqueous solution of 1.76 mM PEG‐Az_4_ (10 kDa, PEGworks, USA). Conductive hydrogels were formed by mixing HA‐BCN with an aqueous solution of 1.76 mM PEG‐Az_4_, an equal amount of ETE‐S and HRP with different concentrations of 0, 5, 10, 20, and 40 mg ml^−1^ in PDMS molds. During cross‐linking, the hydrogels were sandwiched between two glass slides covered with parafilm and kept moist and incubated at room temperature for 30 min. The resulting hydrogels were then treated with 1 mL H_2_O_2_ solution (1 mM in PBS) for at least 2 h at room temperature to form the conductive polymers inside the hydrogel, and all hydrogel samples were kept in PBS buffer for further characterization. One more step of freeze drying was performed for hydrogel sample preparation for SEM analysis.

##### SEM Imaging of Material Structures

Freeze‐dried HA‐BCN/PEG hydrogels with 0, 5, 10, and 20 mg ml^−1^ PETE‐S were studied using a JEOL 7800 F Prime SEM at an acceleration voltage of 1 kV at high vacuum conditions. The nonconductive HA‐BCN/PEG hydrogel with 0 mg ml^−1^ PETE‐S was sputter coated with a 5 nm gold film to prevent charging during imaging in the SEM. The hydrogels with added PETE‐S polymer were conductive and did not require sputter coating. Information about the surface morphology was obtained using the secondary electron signal for imaging.

##### Focused Ion Beam (FIB) Milling to Reveal the Internal Microstructure

Freeze‐dried HA‐BCN/PEG hydrogels with 0, 5, 10, and 20 mg ml^−1^ PETE‐S were cross‐sectioned using FEI Versa3D and Tescan GAIA3 FIB‐SEM instruments to reveal the internal and subsurface microstructure of the materials. The hydrogels were sputter coated with a thick layer of gold to enhance the conductivity of the specimen surface. An additional layer of platinum was deposited in the FIB‐SEM instruments before milling in order to protect the surface structure as previously described for soft, porous, and poorly conducting materials.^[^ ^31^ ^]^

##### Rheological Assessment of Hydrogels

Hydrogel disks, 8 mm in diameter, were used for rheological characterization (*n* = 3 for each condition) and analyzed using a TA instrument HR‐2 oscillatory rheometer (TA instruments, New Castle, USA). Frequency sweeps (0.1–10 Hz and 1% strain) and amplitude sweeps (0.1%–50% strain, 1 Hz) were conducted using an 8 mm parallel geometry at room temperature for both nonconductive hydrogels and conductive hydrogels treated with H_2_O_2_ or PBS for at least 2 h. The time sweeps study was conducted in the linear viscoelastic region for 300 s.

##### Evaluation of Cytocompatibility of ETE‐S Using Human Primary Fibroblasts

All experiments involving human tissue and cells were performed under ethical approval from the Swedish Ethical Review Authority (no. 2018/97‐31) and following ethical standards postulated by Linköping University and Swedish and European regulations. Human primary dermal fibroblasts were collected from skin biopsies of healthy individuals (Passage 5) and subsequently cultured in a culture flask containing DMEM F12 medium supplemented with 10% FBS until they attained a 70% confluent state. The cells were then trypsinized and counted, and 12 000 cells were plated in each well of a 96‐well plate 1 day before the experiment to allow for cell attachment. To test the effect of ETE‐S on cell viability, ETE‐S was dissolved in culture medium (5 mg ml^−1^) and added to each well, followed by incubation for varying time periods (30, 60, 120, and 240 min) before washing with fresh medium. Cell viability was assessed using live/dead staining, whereby samples were treated with 2 μM calcein AM and 4 μM ethidium homodimer‐1 for at least 20 min at 37 °C. Finally, the samples were imaged using a confocal microscope (Zeiss LSM 780, Carl Zeiss, Oberkochen, Germany).

##### Evaluation of Cytocompatibility of ETE‐S Using PC12 Cells

PC12 cells, derived from rat pheochromocytoma, have undergone thorough investigation, rendering them well‐defined as a neuronal model. PC12 cells (purchased from Sigma) were cultured in flasks with a growth medium made of RPMI 1640 supplemented with 10% FBS and 5% HS. Cell viability and cell density were quantified by erythrosine B staining before each experiment. A 96‐well plate was first coated with 0.01% PLL prepared in distilled water for 10 min followed by PBS buffer solution (10 mM, pH 7.4) rinsing before seeding PC12 cells (20 000 cells per well). ETE‐S monomer solutions with concentrations of 0, 1, 5, 10, 20, and 40 mg ml^−1^ prepared in RPMI 1640 were used to replace the growth medium after cell seeding in 12 h and were incubated with seeded PC12 cells for 30 min in a 96‐well plate. The cytotoxic effects of ETE‐S monomers on PC12 cells were assessed using a fluorescent live/dead staining kit with 2 μM calcein AM and 4 μM ethidium homodimer‐1 treated for 30 min at 37 °C. The stained cells were imaged using the Zoe fluorescent cell imager (Bio‐Rad Laboratories). Each ETE‐S concentration sample was analyzed with three replicates and two technical replicates. Statistics analysis was performed employing ordinary one‐way analysis of variance (ANOVA), followed by Dunnett's multiple comparisons test. The p‐values were adjusted for multiple comparisons.

##### Evaluation of Cytocompatibility of Conductive Hydrogels Using PC12 Cells

To test the biocompatibility of ETE‐S containing HA‐BCN/PEG hydrogels and the effect of H_2_O_2_ addition when triggering the enzymatic polymerization in the presence of PC12 cells, a hydrogel precursor solution containing HA‐BCN, PEG‐Az_4_, ETE‐S, and HRP was mixed with PC12 cells to create a cell‐laden hydrogel with a cell density of 1000 cells μL^−1^. 10 μL of the cell‐laden hydrogels were drop‐cast in a 24‐well plate with three replicates. After 30‐min hydrogel cross‐linking at 37 °C, 5% CO_2_ in a humified atmosphere, RPMI 1640 medium was added on top of the hydrogels. Small volumes of a 10 mM H_2_O_2_ solution were added stepwise to the cell‐laden hydrogel in RPMI 1640 medium, to a maximum concentration of 100 μM H_2_O_2_ to initiate mild enzymatic polymerization of ETE‐S in the presence of PC12 cells. Cell proliferation was assessed by an alamarBlue assay (Thermo Fisher Scientific, Waltham, USA) on days 1, 3, and 7. At each time point, cell‐laden hydrogels were initially washed with PBS buffer solution (10 mM, pH 7.4) and then incubated in 10% v/v alamarBlue reagent solution prepared in RPMI 1640 medium for 3.5 h. In order to eliminate potential contamination contributing to the fluorescence intensity, the supernatant of cell‐laden hydrogels was first collected and transferred to a 96‐well plate followed by incubating with 10% v/v alamarBlue reagent solution for 3.5 h on day 7. Fluorescence intensity was measured using an excitation wavelength of 560 nm and an emission wavelength of 590 nm. Each group of cell‐laden gel samples was analyzed with four to nine replicates and was evaluated by relative cell proliferation ratio between day 7 and day 1. Statistical analysis was performed using ordinary one‐way ANOVA, followed by Dunnett's multiple comparisons test. The p‐values were adjusted for multiple comparisons.

##### Characterization of Electrical Properties

HA‐BCN/PEG hydrogel precursor solutions containing equal amounts of ETE‐S and HRP at concentrations of 0, 10, and 20 mg ml^−1^ were drop‐cast on top of AuNW electrodes inside PDMS wells (*D* = 4 mm). The HA hydrogel precursor solution started to gel directly after mixing with PEG‐Az_4_ and was fully gelled within 30 min at room temperature. After adding H_2_O_2_ solution to the preformed hydrogel, the embedded ETE‐S started polymerization in the presence of HRP enzyme, and the hydrogel color turned from light brown to dark blue. Electrochemical impedance spectroscopy (EIS) measurements were performed using a Gamry potentiostat in a three‐electrode setup with a Ag/AgCl pellet as reference electrode and a platinum wire as counter electrode in PBS buffer solution (10 mM, pH 7.4) as supporting electrolyte. AuNW working electrodes and conductive hydrogel‐coated AuNW working electrodes with identical dimensions (*D* = 4 mm) were used. EIS was performed in a frequency range between 20 kHz and 0.1 Hz at *V*_DC_ = 0 V versus *V*_Ref_ with an AC amplitude of 10 mV. Cyclic voltammetry was performed using the same setup and the same supporting electrolyte for EIS measurements. Samples were measured over the potential range from –0.1 to 0.1 V at a scan rate of 50 mV s^−1^. To monitor the enzymatic polymerization of ETE‐S monomers inside the hydrogel matrix, a voltage of 0.1 V, lower than the electropolymerization potential for ETE‐S monomers, was applied between two adjacent AuNW electrodes with a spacing distance of 150 μm.

Additionally, the electrical characteristics of the hydrogels were studied using planar Au electrodes to measure the material's resistance after the incorporation and polymerization of ETE‐S. The measurements were performed using four parallel Au lines spaced 10 μm apart, and the film was in the dried state. The voltage difference between the two inner probes was recorded while applying a constant current between two external probes.

##### Printing HA‐BCN/PEG/ETE‐S Hydrogel Ink

A solution of HA‐BCN/PEG/ETE‐S was prepared as described earlier. PEG‐Az_4_ was added 5 min before printing to make the solution more viscous. Printing was conducted with a BIO X 3D bioprinter (CELLINK, Sweden) with a 25 G printing nozzle. The pressure was set to 20 kPa, and the nozzle moved at a speed of 10 mm s^−1^. A single‐layer lattice (10 × 10 × 0.3 mm) was printed and kept hydrated for 30 min to allow for sufficient cross‐linking before completely covering it with PBS buffer solution (10 mM, pH 7.4). Samples were treated with 1 mM H_2_O_2_ after gelation to initiate the polymerization of ETE‐S for 2 h.

##### Printing in Gelatin Support Bath

A thermo‐reversible gelatin support bath was prepared as previously described.^[^ ^37^ ^]^ Briefly, a solution of gelatin 4.5% w/v was prepared in MilliQ water and blended using a mixer to form a slurry. After washing with cold PBS buffer solution (10 mM, pH 7.4), the slurry was centrifuged at 4000 rpm and 4 °C before being used for printing. The HA‐BCN/ETE‐S solution was prepared as described earlier. PEG‐Az_4_ was added right before printing started. The material was printed by 3 kPa pressure and 10 mm s^−1^ nozzle speed into a 5 × 5 × 1 mm lattice. The printed structure was kept in the support bath at room temperature for at least 1 h before the gelatin slurry was melted at 37 °C and washed away by warm PBS buffer solution (10 mM, pH 7.4). The washed structure was then treated with 1 mM H_2_O_2_ for 2 h.

##### Bioprinting of PC12 Cells Using HA‐BCN/PEG/ETE‐S Hydrogel Bioink

A precursor solution containing HA‐BCN, 1 mg ml^−1^ ETE‐S, and 1 mg ml^−1^ HRP was mixed and sterilized by UV light. PC12 cells suspended in RPMI 1640 medium were mixed gently with 100 μL precursor solution followed by the addition of PEG‐Az_4_ solution to obtain a final cell density of 1000 cells μL^−1^. The prepared bioink was transferred to a 25‐gauge needle and connected to a bioprinter printing head. A single‐layer lattice (10 × 10 ×0.3 mm) was printed and was kept hydrated for 30 min by storing it in a sealed and moisture‐rich environment to allow for sufficient cross‐linking before completely covering it with RPMI 1640 culture solution. Small volumes of a 10 mM H_2_O_2_ solution were added stepwise to the bioprinted hydrogel in RPMI 1640 medium, to a maximum concentration of 100 μM H_2_O_2_ to initiate mild enzymatic polymerization of ETE‐S in the presence of PC12 cells. Cell viabilities after bioprinting were compared to the samples prepared with the same bioink using another deposition method via pipetting and assessed by live/dead assay on day 1.

##### Statistical Analysis

Statistical analysis was carried out using GraphPad Prism 9 software (San Diego, CA, USA). The specific data analysis used for each data set is mentioned in the figure captions.

## Conflict of Interest

The authors declare no conflict of interest.

## Author Contributions

**Changbai Li**: Conceptualization (supporting); formal analysis (lead); investigation (lead); methodology (equal); validation (supporting); visualization (lead); writing—original draft (lead); writing—review and editing (equal). **Sajjad Naeimipour**: Conceptualization (supporting); formal analysis (lead); investigation (lead); methodology (lead); validation (equal); visualization (equal); writing—original draft (lead); writing—review and editing (equal). **Fatemeh Rasti Boroojeni**: Investigation (supporting); methodology (supporting). **Tobias Abrahamsson**: Investigation (supporting); methodology (supporting); resources (lead). **Xenofon Strakosas**: Conceptualization (supporting); investigation (supporting); methodology (supporting); supervision (equal); validation (supporting). **Yangpeiqi Yi**: Formal analysis (supporting); investigation (supporting); methodology (supporting). **Rebecka Rilemark**: Investigation (supporting); methodology (supporting). **Caroline Lindholm**: Conceptualization (supporting); supervision (supporting); validation (supporting); visualization (supporting). **Venkata Perla**: investigation (supporting); methodology (supporting). **Chiara Musumeci**: Methodology (supporting); supervision (supporting); validation (supporting). **Yuyang Li**: Investigation (supporting); methodology (supporting). **Hanne Biesmans**: Investigation (supporting); methodology (supporting). **Marios Savvakis**: Investigation (supporting); methodology (supporting). **Eva Olsson**: Funding acquisition (supporting); resources (supporting); validation (supporting). **Klas Tybrandt**: Conceptualization (supporting); funding acquisition (supporting); supervision (supporting); validation (supporting). **Mary J Donahue**: Methodology (supporting); supervision (supporting); validation (supporting). **Jennifer Y Gerasimov**: Conceptualization (supporting); methodology (supporting); supervision (equal); validation (supporting). **Robert Selegård**: Investigation (supporting); methodology (supporting); supervision (supporting). **Magnus Berggren**: Conceptualization (supporting); funding acquisition (lead); resources (lead); supervision (supporting). **Daniel Aili**: Conceptualization (equal); funding acquisition (lead); project administration (lead); resources (lead); supervision (lead); validation (lead). **Daniel Simon**: Conceptualization (lead); funding acquisition (lead); project administration (lead); resources (lead); supervision (lead); validation (lead); writing—original draft (supporting); writing—review and editing (equal). **Changbai Li** and **Sajjad Naeimipour** contributed equally to this work.

## Supporting information

Supplementary Material

## Data Availability

The data that support the findings of this study are available from the corresponding author upon reasonable request.
