# Supplementary material for: Engineering Conductive Hydrogels with Tissue‐like Properties: A 3D Bioprinting and Enzymatic Polymerization Approach
Source: Small Sci. 2024 Sep 1;4(11):2400290. doi: 10.1002/smsc.202400290 (PMC11935014; doi:10.1002/smsc.202400290)
Supplement: Supplementary file 1 — Supplementary Material [file SMSC-4-2400290-s001.pdf]

SUPPLEMENTARY INFORMATION

Engineering conductive hydrogels with tissue-like properties: a 3D bioprinting and enzymatic polymerization approach

Changbai Li[1], Sajjad Naeimipour[2], Fatemeh Rasti Boroojeni[2], Tobias Abrahamsson[1], Xenofon Strakosas[1], Yangpeiqi Yi[1], Rebecka Rilemark[3], Caroline Lindholm[1], Venkata K. Perla[1], Chiara Musumeci[1], Yuyang Li[1], Hanne Biesmans[1], Marios Savvakis[1], Eva Olsson[3], Klas Tybrandt[1], Mary J. Donahue[1,4], Jennifer Y. Gerasimov[1], Robert Selegård[2], Magnus Berggren[1], Daniel Aili[2,*], Daniel T. Simon[1,*]

[1] Laboratory of Organic Electronics, Dept. Science and Technology, Linköping University, 601 74 Norrköping, Sweden

[2] Laboratory of Molecular Materials, Division of Biophysics and Bioengineering, Department of Physics, Chemistry and Biology, Linköping University, 581 83 Linköping, Sweden

[3] Department of Physics, Chalmers University of Technology, 41296, Göteborg, Sweden

[4] Bioelectronics Materials and Devices Lab, Central European Institute of Technology, Brno University of Technology, Purkyňova 123, 61200 Brno, Czech Republic.

* Corresponding authors: daniel.aili@liu.se, daniel.simon@liu.se

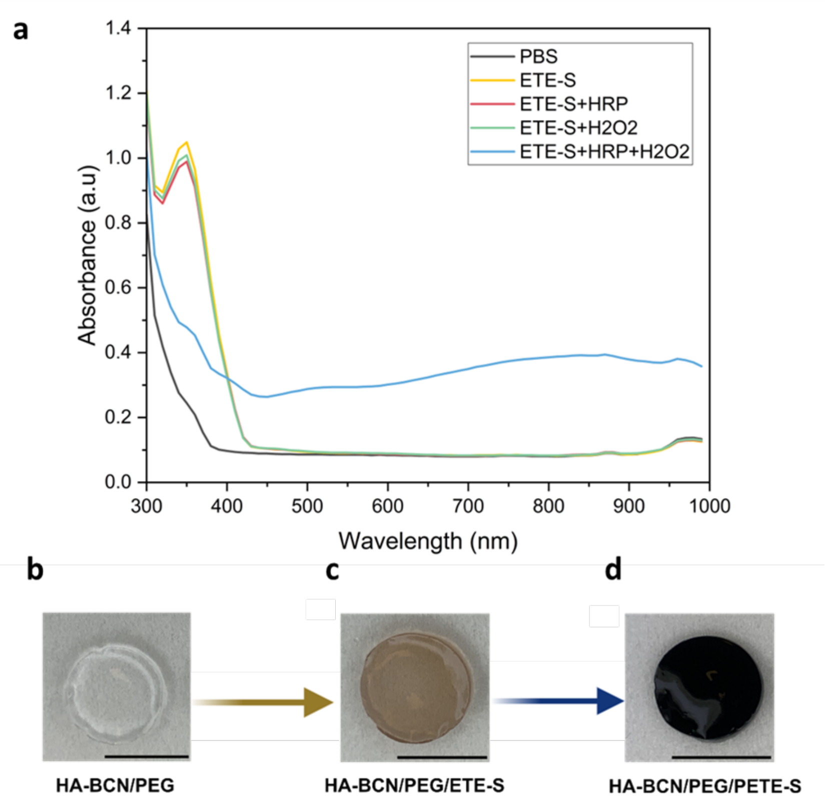

Figure S1. Absorption spectra of a) PBS buffer solution (10 mM, pH 7.4); ETE-S (0.1 mg/ml, 176 µM); ETE-S (0.1 mg/ml, 176 µM) with HRP (0.1 mg/ml); ETE-S (0.1 mg/ml, 176 µM) with H_2O_2 (265 µM); and ETE-S (0.1 mg/ml, 176 µM) with both HRP (0.1 mg/ml) and H_2O_2 (265 µM) in PBS buffer solutions (10 mM, pH 7.4) (n=1). Photograph of b) the transparent HA-BCN/PEG hydrogel, and c) the light brown HA-BCN/PEG hydrogel containing 5 mg/ml ETE-S monomers and HRP enzyme after 2h of swelling in PBS buffer solution at room temperature. d) Photograph of the HA-BCN/PEG hydrogel containing 5 mg/ml ETE-S monomers and HRP enzyme after treating with H_2O_2 for 2h at room temperature with characteristic dark blue PETE-S, i.e., “PETE-S-5 gel”. (scale bars = 8 mm)

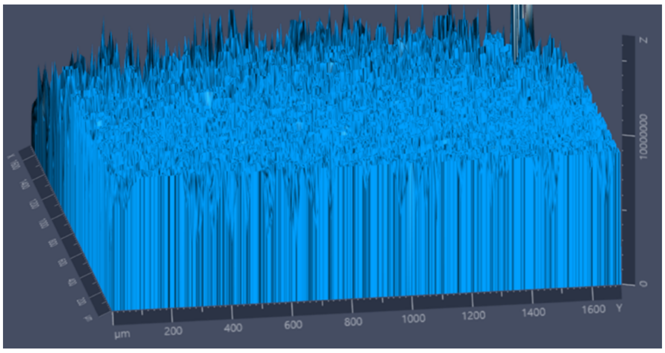

Figure S2. Confocal fluorescence image of 5 mg/ml ETE-S gel. Blue fluorescence (DAPI filter) indicates the presence of ETE-S monomers before polymerization through hydrogel matrix (unit for x-axis and y-axis: μm and unit for z-axis: fluorescence intensity a.u.).

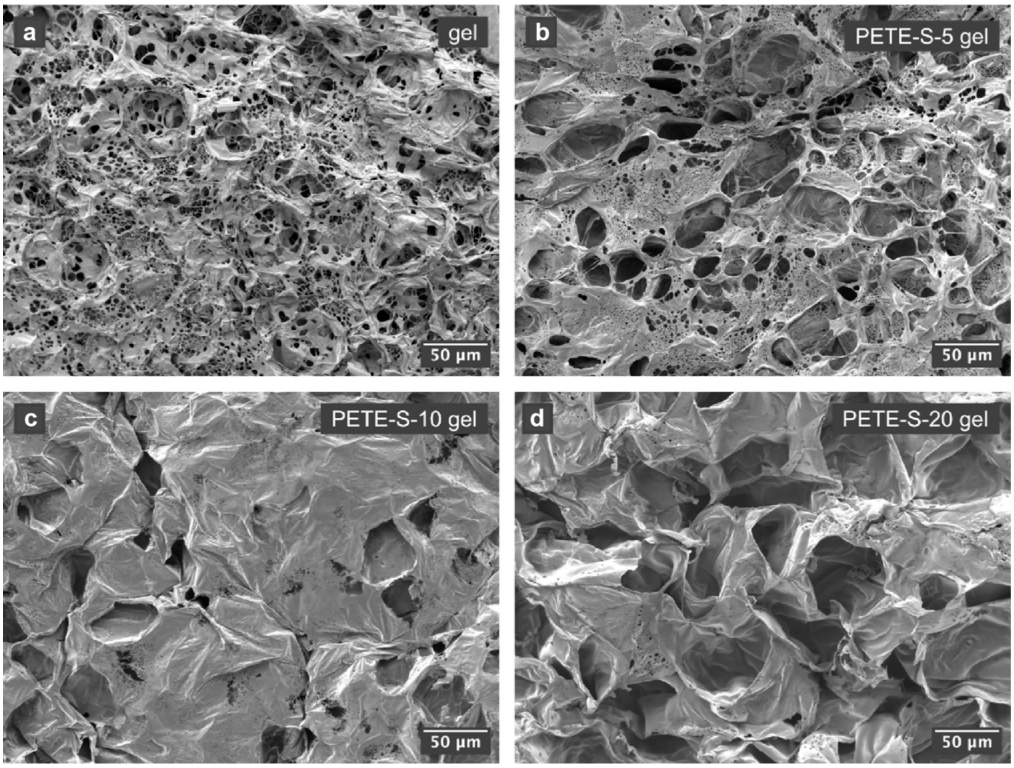

Figure S3. a) SEM secondary electron images of freeze-dried hydrogel samples showing the surface morphology of clean gel (no PETE-S), (b) PETE-S-5 gel, (c) PETE-S-10 gel, and (d) PETE-S-20 gel. The structure consists of pores separated by walls. The walls become more continuous as the PETE-S concentration increases.

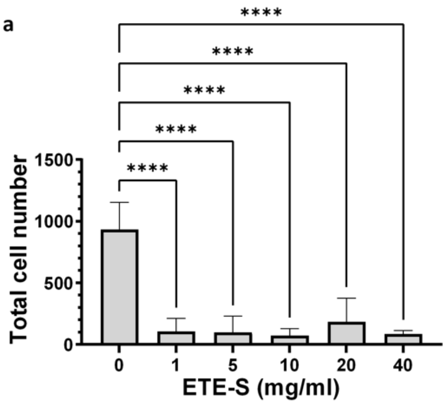

Figure S4. a) Total cell numbers of PC12 cells evaluated by live/dead assay after 30-min incubation with 0, 1, 5, 10, 20, and 40 mg/ml ETE-S, One-way ANOVA analysis and Dunnett's multiple comparisons test were used, **** P < 0.0001 (n = 6 replicates for each concentration).

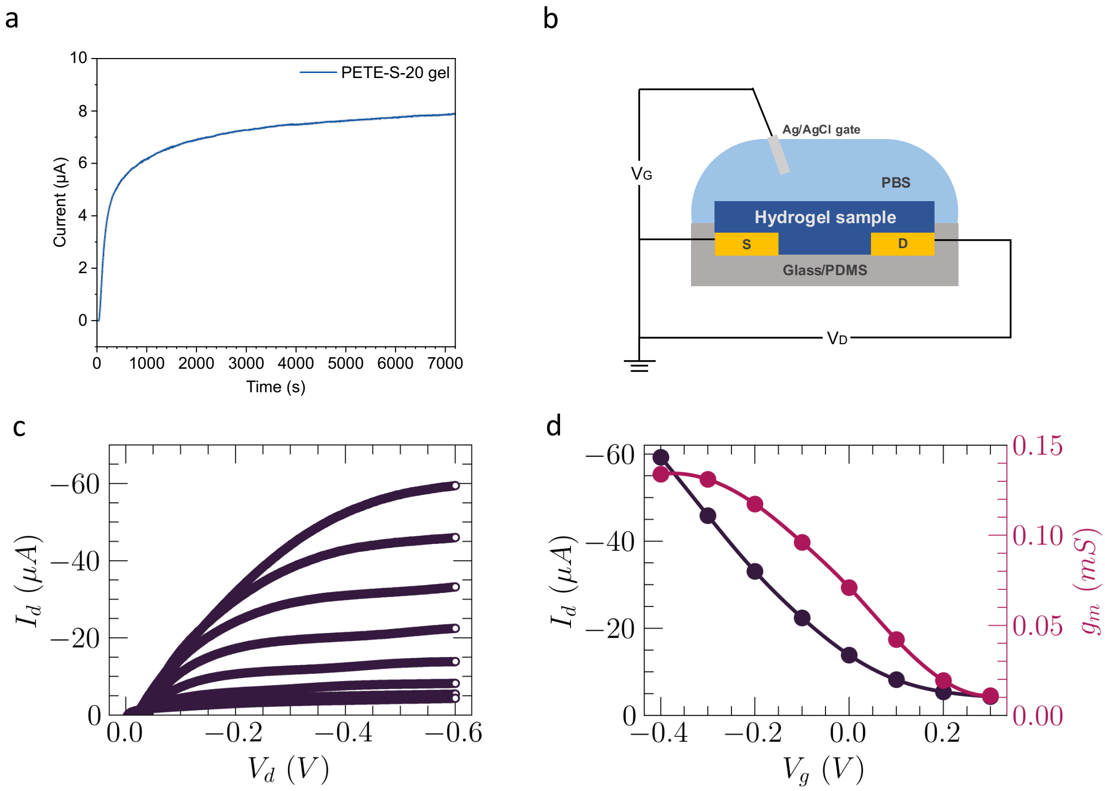

Figure S5. a) Current versus time measurement during the polymerization of HA-BCN/PEG/ETE-S hydrogel after the addition of H_2O_2. The HA-BCN/PEG/ETE-S hydrogel was prepared in a mixed solution where 20 mg/ml HA-BCN and 1.76 mM PEG-Az4 were mixed with 20 mg/ml ETE-S monomer and 20 mg/ml HRP. The increased current between two parallel AuNW electrodes (spacing distance = 150 μm) during applied constant voltage 0.1 V indicated that the addition of H_2O_2 (t = 0 s) initiated the enzymatic polymerization. The increased current followed a sigmoidal behavior and its peak magnitude showed that the completion of enzymatic polymerization (t = 7200 s; n=1 individual measurement). b) Schematic of OECT with circuit connections representing the measurement setup. c) Output characteristics (I_D vs V_D) of PETE-S-20 gel based OECT device (L = 150 μm, W = 4000 μm) for gate voltage (V_G) varying from -0.4 V (top curve) to + 0.3 V (bottom curve) with a step of + 0.1 V (I_D, V_D, and V_G notate drain current, drain voltage, and gate voltage,

n=1). d) Transfer curve for V_D = − 0.6 V (black), and the corresponding transconductance (red), $|g_{m,max}|$ = 0.13 mS (n=1).

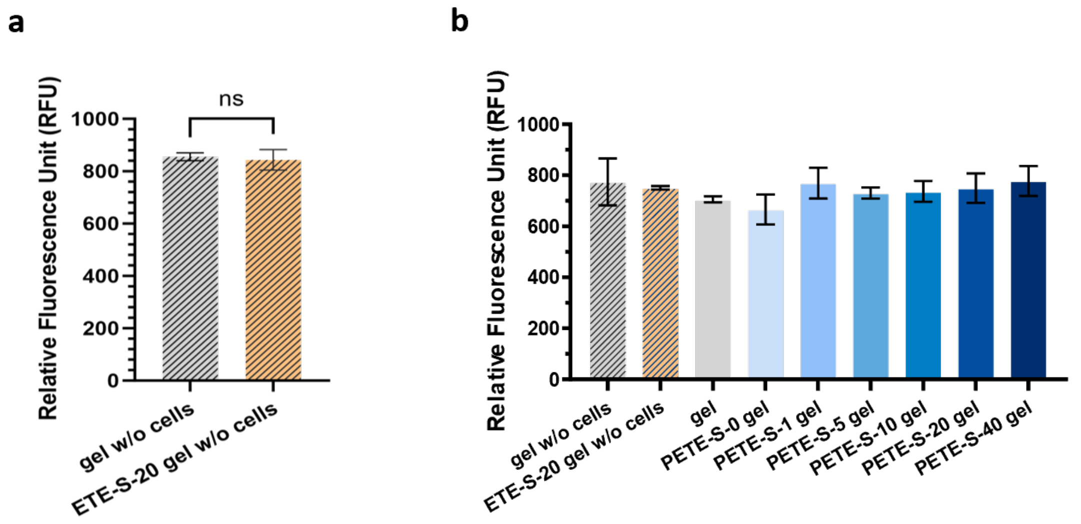

Figure S6. a) Alamar Blue assay incubated with gel without PC12 cells (grey), and gel without PC12 cells incorporated with 20 mg/ml ETE-S monomers (orange) on day 1 (Unpaired t-test, n = 4 replicates for each group, not significant). b) Solution contamination check for unexpected interferents via Alamar Blue assay on day 7 (n = 2 replicates for each group), One-way ANOVA with Tukey's multiple comparisons test, not significant.
